# Approach and solutions to congenital hearing impairment in Cameroon: perspective of hearing professionals

**DOI:** 10.1186/s41182-022-00430-7

**Published:** 2022-05-30

**Authors:** Emmanuel Choffor-Nchinda, Jean Valentin Fokouo Fogha, Adèle-Rose Ngo Nyeki, Asmaou Bouba Dalil, Roger Christian Meva’a Biouélé, Geschiere Peter Me-Meke

**Affiliations:** 1 Department of Surgery and Specialties, Faculty of Health Sciences, University of Buea, PO Box 63, Buea, Cameroon; 2 COCHLEES Research Group, Yaoundé, Cameroon; 3 ENT Unit, Bertoua Regional Hospital, Bertoua, Cameroon; 4 Department of Ophthalmology, Otolaryngology and Stomatology, Faculty of Medicine and Biomedical Sciences, University of Yaoundé I, PO Box 1364, Yaoundé, Cameroon; 5 ENT Unit, Limbe Regional Hospital, Limbe, Cameroon

**Keywords:** Congenital, Hearing loss, Sub-Saharan Africa, Neonatal screening, Cameroon

## Abstract

**Objectives:**

To bring out the diagnostic attitude of hearing professionals in Cameroon towards congenital hearing impairment (CHI), assess availability of tests, neonatal screening, and create a national map of availability of treatment opportunities.

**Methods:**

We conducted a cross-sectional online-based survey from June to December 2021, concerning ear–nose–throat (ENT) specialists, hearing care professionals, speech therapists and ENT nurses. A Google Forms online questionnaire was used to collect data, filled by eligible professionals involved in hearing care in Cameroon.

**Results:**

A total of 93 professionals working in 31 different health facilities participated. A cumulative percentage of 79.9% of ENTs were found in just two out of 10 regions. Specialists sought by ENTs for assessment of patients with CHI included neurologists/neuro-pediatricians (96.8%), pediatricians (47.6%), other ENTs (34.9%), and psychologists (3.2%). Investigations requested included auditory-evoked brainstem response (ABR; 87.3%), otoacoustic emissions recording (OAE; 71.4%), and tympanometry (66.7%). There were eight OAE and nine ABR machines in the country. Twenty-five (88.6%) out of 31 facilities with otolaryngologists did not carry out systematic neonatal screening. Reasons included unavailability of equipment (21; 84%), and administrative delays (14; 56%). Sixteen (51.6%) facilities had ENTs with additional training in otologic surgery and 11 (35.5%) were equipped to perform ear surgery. Three centers (9.7%) specialized in hearing aid provision and maintenance services. Three hospitals (9.7%) had performed cochlear implantation.

**Conclusion:**

Our results show scarcity and overt unevenness in distribution of specialists, equipment and solutions to CHI in Cameroon. A serious negative health care consequence of this shortage is the unavailability of universal newborn hearing screening and implementation programs.

## Background

Congenital hearing impairment (CHI) remains a major public health problem in sub-Saharan Africa (SSA). According to the World Health Organization (WHO), an estimated 466 million people worldwide have disabling hearing impairment (HI), among whom 49 million live in SSA and 34 million are children [1]. CHI affects up to 6 per 1000 live births in SSA compared with about 1 per 1000 live births in high income countries [2, 3]. Etiological factors of CHI include genetic factors (30–50%) [4] as well as prenatal, perinatal and postnatal factors resulting from infections, asphyxia, hyperbilirubinemia and ototoxicity [5]. The non-genetic factors representing about 70% of CHI are preventable [1] and are bolstered in SSA by high prevalence of infections, malnutrition, poverty and poor access to health care.

Much of the impact of HI can be mitigated through early detection and interventions [1]; management involves early identification, proper characterization of the defect, treatment, rehabilitation and possible prevention, leading to improved developmental outcomes later in childhood [6]. In most high income countries, neonatal hearing screening programs are available to detect and manage this condition. These programs aim to screen all newborns within one month of birth [6]. They also provide a wide variety of social services, accessibility enhancement and rehabilitation programs.

In SSA, HI is usually given less priority despite its significant burden [7]. There is generally lack of information and awareness about CHI, insufficient human and material resources, and poor accessibility to treatment. Furthermore, neonatal screening programs are scarce to non-existent, and as a result many children are missed at the stage when adequate interventions would be most helpful.

The objective of this study was to bring out the diagnostic attitude of hearing professionals in Cameroon towards CHI, assess availability and distribution of neonatal screening programs, related tests, and treatment possibilities. This would raise awareness among practitioners and policy-makers about hearing care in Cameroon, enabling possible improvements in policies and the general approach to CHI. This study would also provide data for the creation of a national map of availability of investigations and treatment opportunities, facilitating referrals among physicians.

## Methods

### Study design and procedure

We conducted a cross-sectional online-based survey from June to December 2021, concerning ear–nose–throat (ENT) specialists (otolaryngologists), hearing care professionals (including audiologists and technicians trained in fitting, adjustment and maintenance of hearing aids), speech therapists and ENT nurses. The survey was carried out with the help of a pre-tested Google Forms® (Mountain View, CA) questionnaire designed to be filled online by all consenting professionals involved in hearing care in Cameroon, irrespective of age or sex. We excluded non-permanent professionals, for instance those working in Cameroon in the context of a fellowship or a cooperation agreement because of the temporary and inconstant nature of their services. Any questionnaire with unclear or incomplete responses was equally excluded when clarification was impossible despite contacting the concerned participant. The questions were written in English and French, the two official languages, in clear and simple medical terms. Compulsory field settings were used for essential questions, while optional question types were used for less important questions or those requiring some explanations. Rapid and effective dissemination of the questionnaire was ensured with the help of various social media groups that comprised hearing professionals. Following this, members of these groups were individually reminded by phone or e-mail to maximize study completion. Contacts and response rates were obtained by consulting the latest version of the national directory of hearing specialists designed by the Cameroonian Society of Otolaryngology-Head and Neck Surgery. Disparities in information provided by participants working in the same health facility were resolved by calling the concerned workers. Answers recorded were exported in a Microsoft Excel® (Redmond, WA) format for analysis.

### Statistics

Answers to the following items obtained from participants comprised variables; socio-demographic data (age, sex, administrative region and town of practice), professional information (specialty, health facility of practice, number of professionals per facility), approach and attitude (systematic neonatal screening, mode of contact with potential patients with CHI, investigations requested, referrals or outside opinions sought), availability of diagnostic and investigative equipment in health facilities according to administrative region (ENT workstation, otologic operating microscope, otoacoustic emissions (OAE), auditory-evoked brainstem response (ABR), behavioral audiometry, pure tone audiometry (PTA), computerized tomography (CT) scan, magnetic resonance imaging (MRI), genetic workup), availability of treatment opportunities (specialists, hearing aids, ear surgery including cochlear implantation, speech therapy, and maintenance services for hearing aids and cochlear implants). Data were analyzed using STATA® version 13 (College Station, TX). Results were presented as mean for quantitative variables, and percentages for qualitative variables.

### Ethical considerations

The survey comprised details on the objectives of the study, benefits of the results and intended use of information collected. A checkbox was used for informed consent, to be ticked by participants prior to filling the questionnaire. Information about participants was kept confidential by replacing names with assigned codes. Ethical clearance to carry out this study was granted by the Institutional Review Board of the Faculty of Health Sciences, University of Buea, Cameroon (Ref: 2021/1398B-11/UB/SG/IRB/FHS).

## Results

### Distribution of professionals

A total of 93 professionals of various hearing-related specialties working in 31 different health facilities across the country participated in this study. Ages varied from 28 to 69 years (mean = 42 ± 8.1 years), with male predominance (54.8%). All 10 regions of Cameroon had specialists. A cumulative percentage of 79.9% of ENTs were found in the Center and Littoral regions. We obtained responses from 63 ENTs (response rate 70.8%), two speech therapists (response rate 66.7%), seven hearing care professionals (response rate 70%) and 21 ENT nurses (response rate 65.6%). Distribution of specialists according to region and specialty based on data collected in this study is represented on Table 1.

**Table 1:** Distribution of hearing professionals in Cameroon by administrative region

Region	ENTs	ENT nurse	HCP	ST
	*N* = 89 (%)	*N* = 32 (%)	*N* = 10 (%)	*N* = 3 (%)
Center	43 (48.3)	11 (34.4)	4 (40)	2 (66.7)
Littoral	28 (31.6)	18 (56.2)	6 (60)	1 (33.3)
South-West	3 (3.4)	0 (0)	0 (0)	0 (0)
North-West	3 (3.4)	0 (0)	0 (0)	0 (0)
West	3 (3.4)	0 (0)	0 (0)	0 (0)
South	2 (2.2)	2 (6.3)	0 (0)	0 (0)
East	2 (2.2)	0 (0)	0 (0)	0 (0)
North	2 (2.2)	1 (3.1)	0 (0)	0 (0)
Adamawa	2 (2.2)	0 (0)	0 (0)	0 (0)
Far North	1 (1.1)	0 (0)	0 (0)	0 (0)

*ENTs* ear–nose–throat specialist, *HCP* hearing care professional, *ST* speech therapist

### Attitude, diagnosis and investigations

Otolaryngologists received most of the children suspected of having hearing loss as direct consultations in 61.9% as opposed to referrals from other specialists in 38.1% of cases. Concerning referrals, a proportion of 69.8% of ENTs received patients mostly from pediatricians, 15.9% mostly from neuro-pediatricians and 14.3% from general practitioners. Other specialists sought by ENTs for an opinion and/or team management included neurologists/neuro-pediatricians (96.8%), pediatricians (47.6%), other ENTs (34.9%), psychologists (3.2%), and ophthalmologists (1.6%). Investigations requested by ENTs included ABR (87.3%), OAE (71.4%), tympanometry (66.7%), CT scan (30.1%), and MRI (3.2%). Table 2 represents the number and distribution of diagnostic equipment units across the country, by administrative region.

**Table 2:** Distribution of facilities with diagnostic equipment for hearing loss by administrative region

Region	ENTWS	OM	OAE	ABR	BA	PTA + T	CT	MRI	GT	UNS
	*N* = 19	*N* = 10	*N* = 8	*N* = 9	*N* = 1	*N* = 18	*N* = 15	*N* = 4	*N* = 0	*N* = 6
Adamawa	0	0	0	0	0	0	1	0	0	0
Center	11	5	5	5	0	9	7	1	0	4
East	0	1	0	0	0	0	0	0	0	0
Far North	0	0	0	0	0	0	0	0	0	0
Littoral	4	3	2	2	1	3	3	2	0	2
North	3	0	1	2	0	3	1	0	0	0
NW	0	0	0	0	0	0	1	0	0	0
South	1	1	0	0	0	2	0	0	0	0
SW	0	0	0	0	0	0	1	0	0	0
West	0	0	0	0	0	1	1	1	0	0

*ABR* auditory-evoked brainstem response, *BA* behavioral audiometry, *CT* computerized tomography scan, *ENTWS* ear–nose–throat workstation, *GT* genetic testing, *MRI* magnetic resonance imaging, *NW* North-West, *OAE* otoacoustic emissions, *OM* operating microscope, *PTA + T* pure tone audiometry with tympanometry, *SW* South-West, *UNS* universal neonatal hearing screening

Twenty-five (88.6%) out of 31 facilities with otolaryngologists did not carry out systematic neonatal screening. Reasons for not implementing screening included unavailability of equipment (21; 84%), implementation pending and delayed due to administrative reasons (14; 56%), and not applicable due to nature of facility (1; 4%).

### Management opportunities

**Fig. 1:**
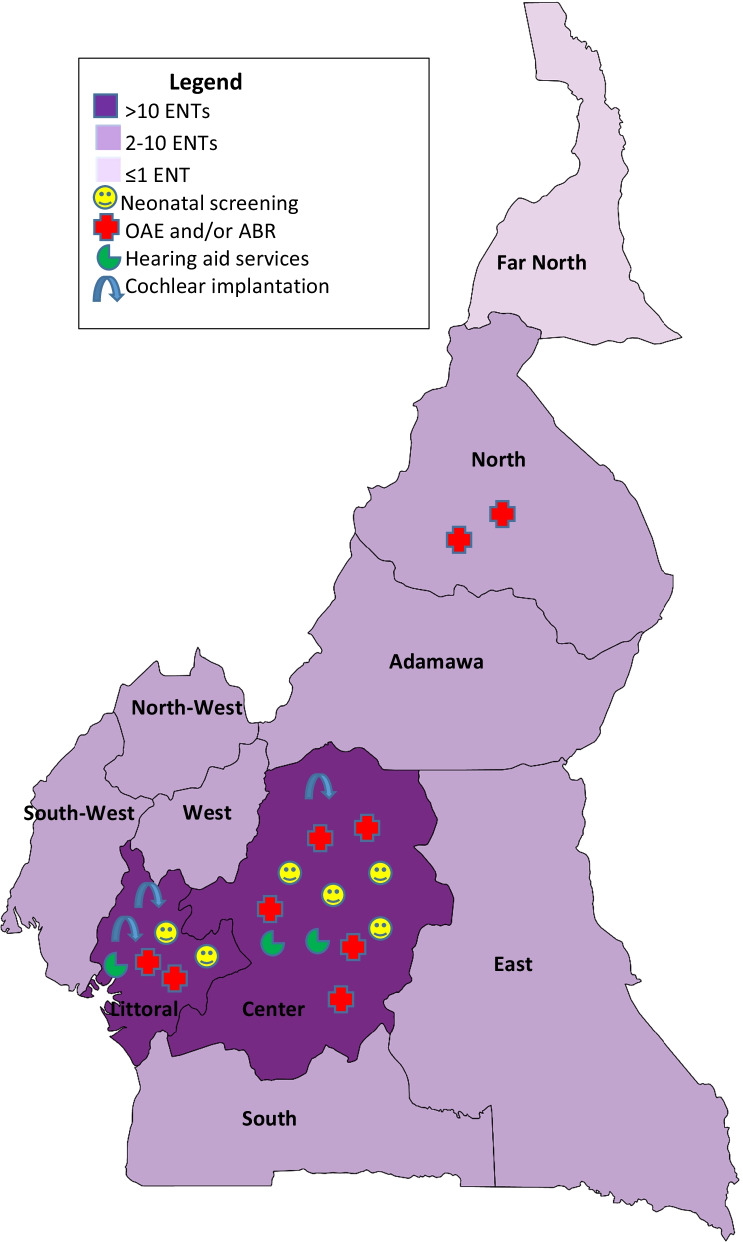
Distribution of specialists and hospitals offering diagnosis and treatment for congenital hearing loss in Cameroon. *ABR* auditory-evoked brainstem response, *ENT* ear–nose–throat specialist, *OAE* otoacoustic emissions

Sixteen (51.6%) facilities had ENTs with some additional training in otology/otologic surgery and 11 (35.5%) were equipped to perform ear surgery. Six of these (54.5%) were located in the center region, four (36.4%) in the littoral and one in the east. Three centers (9.7%) specialized in hearing aid provision and maintenance services, two in the Center and one in the Littoral regions. Three hospitals performed cochlear implantation, with the possibility of providing maintenance for these implants. Figure 1 represents the distribution of otolaryngologists in Cameroon, availability of screening, basic hearing investigations and treatment possibilities.

## Discussion

Cameroon is a tropical sub-Saharan African country with a surface area of 475,000 km^2^, a population of approximately 27 million and an estimated overall physician–patient ratio of 1 in 50,000 [8]. It is a low-middle income country with a GDP of 40.8 US dollars [9]. Cameroon’s human development index value for 2019 was 0.563 [10], with a life expectancy at birth of 58.35 years [11], and 45% of the population living below the poverty line [12]. The country is partitioned into 10 administrative regions, each comprising at least one tertiary level hospital with specialists, serving populations varying from about 1 million to over 4 million. All hospitals included in this survey had at least one hearing professional, and all 10 regions had at least an otolaryngologist. There was a gross imbalance in the distribution of specialists; the Center and Littoral regions alone, respectively, containing the political capital (Yaoundé) and economic capital (Douala), accounted for almost 80% of all ENTs (Table 1). This rural–urban disparity exists in almost all of sub-Saharan Africa, where the geographic distribution of health workers favors urban over rural areas [13, 14]. Labor economics [13] and better living conditions in cities could partly explain this unevenness. Similarly, ENT nurses were predominantly found in urban areas, while hearing care professionals and speech therapists were exclusively found in Yaoundé and Douala. This comprises a major challenge and implies that comprehensive care for children with CHI cannot be provided anywhere else.

Contact between the specialist and children with potential CHI was mostly through direct consultations, for various hearing- or speech-related complaints. This is indicative that many children are missed at birth, since these children are brought by their parents who are usually unsure about their children’s hearing status. Other personnel who predominantly intervene in the diagnosis chain included pediatricians, neuro-pediatricians and general practitioners. These categories of physicians generally refer to or seek an opinion from ENTs when faced with possible cases of CHI. In Cameroon, hearing investigations are done by or under the supervision of otolaryngologists. Subsequently, confirmation of suspicion of CHI or any form of hearing disorders invariably passes through them. Interestingly, ENTs generally requested the opinion of other specialists for diagnosis and/or team management, including neurologists/neuro-pediatricians, pediatricians, other ENTs, psychologists, and ophthalmologists. A multidisciplinary approach is indispensable for adequate assessment, including careful prenatal and neonatal history, complete head and neck examination, exhaustive general physical examination, to be complemented by paraclinical tests [15]. This can only be ensured by involving other specialists in the process. In addition, genetic consultation and evaluation is vital. Benefits can include providing etiologic information, identifying comorbidities that may need referral, planning for future medical and educational needs, facilitating estimations of the likelihood of recurrence, allowing families to better plan for the birth of a deaf or hard-of-hearing child, relieving guilt, enhancing psychological well-being, dispelling misinformation, and facilitating referral for unrelated conditions [16–19]. Data from our study reveal unavailability of genetic consultations in Cameroon. This implies that the management of about 30% of these patients [20] will be suboptimal.

The uneven distribution of clinical and paraclinical diagnostic equipment was evident, with a concentration of both hearing test and diagnostic imaging equipment in the Center and Littoral regions (see Table 2). Most importantly, the whole country has only eight OAE machines and nine ABR machines, with seven of each of these machines located in hospitals in Yaoundé and Douala (see Fig. 1). OAE and/or ABR are indispensable for screening and assessment of children with CHI. The acute scarcity of these machines across the country signifies that many children are undiagnosed. This deficiency partly explains why systematic neonatal screening was reported to be implemented only in six health facilities. Universal newborn hearing screening is recommended by WHO [21] and many specialist associations and committees around the world [22]. Without this, diagnosis is invariably delayed and infants with CHI are typically identified with an established language delay [23, 24]. Despite differences in economic and social circumstances, some lower middle income countries such as Nigeria, India, and Philippines [24, 25] have successfully implemented newborn hearing screening programs. Reported obstacles to implementation include the lack of evidence-based local guidance, lack of financial and/or human resources, and the absence of political will [24].

Availability and accessibility to treatment and rehabilitation was yet again problematic, with clustering of the few facilities providing solutions around the economic and political capital cities of the country (see Fig. 1). Interestingly, half of the hospitals had ENTs trained in otologic surgery, though only a few were equipped to perform surgical procedures. A few centers were equipped to supply, set and maintain hearing aids, while three facilities had successfully placed cochlear implants, although the interventions were done in the context of health campaigns with huge support from the government and multinational partners. Likewise, there is an extreme scarcity of speech therapists in Cameroon, which is a cause for concern given the crucial role of speech therapy following hearing rehabilitation [1]. Remarkably, no single institution in Cameroon was able to propose the full treatment package, from otologic surgery, hearing aid supply, cochlear implantation and speech therapy. This trend is similar in most SSA countries, where ENT surgeons, audiologists and especially speech therapists are extremely scarce [25, 26]. The number of available speech therapists in some SSA countries varied from none to six according to a survey done in 2017 [26]. Only South Africa had a distinctively higher quantity of specialists and services available.

This study gives a picture of sites offering hearing-related services for the benefit of patients. An increase in recruitment of specialists, with specific emphasis on genetic consultants and speech therapists, enhanced provision of diagnostic and surgical equipment, and better distribution of the few resources currently available would improve accessibility to hearing care. Implementation of special measures such as incentives for specialists working in semi-urban settings could encourage professional mobility. Implementation of universal newborn screening, treatment and rehabilitation programs would be greatly beneficial.

Limitations of this study include the nature of data collection, making it difficult to verify certain details. This was mitigated by individually contacting participants to clarify discordant information. In addition, though acceptable, survey response rates per professional group were all below 80%, making findings not exhaustive.

## Conclusion

Our results show scarcity and overt unevenness in distribution of specialists, equipment and solutions to CHI in Cameroon. The paucity of essential diagnostic equipment such as OAE and ABR instruments raises concerns about effective diagnosis. There is an imperative need for the implementation of comprehensive hearing management programs, including universal newborn hearing screening, treatment and rehabilitation, which can guarantee a leap in diagnosis and outcome amelioration of CHI. Specialist societies and policy-makers should advocate and institute such programs that have proven to be possible even in resource-limited settings.

## Data Availability

Data sharing is not appropriate for this study. Despite the fact that names were not included, names of towns and hospitals included in the datasets could enable precise identification of respondents, compromising confidentiality.

## Abbreviations

ABR: Auditory-evoked brainstem response
CHI: Congenital hearing impairment
CT: Computerized tomography
ENT: Ear–nose–throat
GDP: Gross domestic product
HI: Hearing impairment
MRI: Magnetic resonance imaging
OAE: Otoacoustic emissions
SSA: Sub-Saharan Africa
WHO: World Health Organization

## References

[1] Deafness and hearing loss. https://www.who.int/westernpacific/health-topics/hearing-loss. Accessed 21 Nov 2021.

[2] Mehra S, Eavey RD, Keamy DG (2009). The epidemiology of hearing impairment in the United States: newborns, children, and adolescents. Otolaryngol Head Neck Surg.

[3] Olusanya BO, Neumann KJ, Saunders JE (2014). The global burden of disabling hearing impairment: a call to action. Bull World Health Organ.

[4] Lebeko K, Bosch J, Noubiap Nzeale JJ, Dandara C, Wonkam A. Genetics of hearing loss in Africans: use of next generation sequencing is the best way forward. https://www.ncbi.nlm.nih.gov/pmc/articles/PMC4499266/. Accessed 21 Nov 2021. 10.11604/pamj.2015.20.383.5230 PMC4499266 26185573

[5] Fraser GR (1964). Profound childhood deafness. J Med Genet.

[6] Korver AMH, Smith RJH, Van Camp G, Schleiss MR, Bitner-Glindzicz MAK, Lustig LR (2017). Congenital hearing loss. Nat Rev Dis Primers.

[7] Adedeji TO, Tobih JE, Sogebi OA, Daniel AD (2015). Management challenges of congenital and early onset childhood hearing loss in a sub-Saharan African country. Int J Pediatr Otorhinolaryngol.

[8] Neba K. Public Health : Over 35000 health care workers needed. Cameroon Radio Television. 2020. https://www.crtv.cm/2020/07/public-health-over-35000-health-care-workers-needed/. Accessed 15 Jan 2022.

[9] World Bank: Cameroon Data. https://data.worldbank.org/country/CM. Accessed 31 Apr 2022.

[10] UNDP: Briefing note for countries on the 2020 Human Development Report. https://hdr.undp.org/sites/default/files/Country-Profiles/CMR.pdf. Accessed 25 Apr 2022.

[11] World Bank: Life expectancy at birth, male (years)—Cameroon. https://data.worldbank.org/indicator/SP.DYN.LE00.MA.IN?locations=CM. Accessed 25 Apr 2022.

[12] World Bank: poverty and equity brief for sub-Saharan Africa—Cameroon. https://databank.worldbank.org/data/download/poverty/33EF03BB-9722-4AE2-ABC7-AA2972D68AFE/Global_POVEQ_CMR.pdf. Accessed 25 Apr 2022.

[13] Lemière C, Herbst CH, Dolea C, Zurn P, Soucat A. Rural-urban imbalance of health workers in sub-Saharan Africa. In: The labor market for health workers in Africa. The World Bank. p. 147–68. (Directions in Development - Human Development). https://elibrary.worldbank.org/doi/abs/10.1596/9780821395554_CH09. Accessed 15 Jan 2022.

[14] Oloyede O. Rural-urban disparities in health and health care in Africa on JSTOR. https://www.jstor.org/stable/90018696. Accessed 15 Jan 2022.

[15] Chen MM, Oghalai JS (2016). Diagnosis and management of congenital sensorineural hearing loss. Curr Treat Options Pediatr.

[16] Palmer CGS, Martinez A, Fox M, Zhou J, Shapiro N, Sininger Y (2009). A prospective, longitudinal study of the impact of GJB2/GJB6 genetic testing on the beliefs and attitudes of parents of deaf and hard-of-hearing infants. Am J Med Genet A.

[17] Brunger JW, Murray GS, O’Riordan M, Matthews AL, Smith RJH, Robin NH (2000). Parental attitudes toward genetic testing for pediatric deafness. Am J Hum Genet.

[18] Burton SK, Withrow K, Arnos KS, Kalfoglou AL, Pandya A (2006). A focus group study of consumer attitudes toward genetic testing and newborn screening for deafness. Genet Med.

[19] Robin NH (2006). It does matter: the importance of making the diagnosis of a genetic syndrome. Curr Opin Pediatr.

[20] Alford RL, Arnos KS, Fox M, Lin JW, Palmer CG, Pandya A (2014). American College of Medical Genetics and Genomics guideline for the clinical evaluation and etiologic diagnosis of hearing loss. Genet Med.

[21] Hearing screening: considerations for implementation. https://www.who.int/publications-detail-redirect/9789240032767. Accessed 23 Apr 2022.

[22] Joint Committee on Infant Hearing, American Academy of Audiology, American Academy of Pediatrics, American Speech-Language-Hearing Association, Directors of Speech and Hearing Programs in State Health and Welfare Agencies. Year 2000 position statement: principles and guidelines for early hearing detection and intervention programs. Joint Committee on Infant Hearing, American Academy of Audiology, American Academy of Pediatrics, American Speech-Language-Hearing Association, and Directors of Speech and Hearing Programs in State Health and Welfare Agencies. Pediatrics. 2000;106(4):798–817. 10.1542/peds.106.4.798 11015525

[23] Patel H, Feldman M (2011). Universal newborn hearing screening. Paediatr Child Health.

[24] Newborn and infant hearing screening. https://www.who.int/blindness/publications/Newborn_and_Infant_Hearing_Screening_Report.pdf?ua=1. Accessed 21 Nov 2021.

[25] Fagan JJ, Jacobs M (2009). Survey of ENT services in Africa: need for a comprehensive intervention. Glob Health Action.

[26] Peer S, Vial I, Numanoglu A, Fagan JJ (2018). What is the availability of services for paediatric ENT surgery and paediatric surgery in Africa?. Eur Ann Otorhinolaryngol Head Neck Dis.

